# Lymphocytes Accelerate Epithelial Tight Junction Assembly: Role of AMP-Activated Protein Kinase (AMPK)

**DOI:** 10.1371/journal.pone.0012343

**Published:** 2010-08-23

**Authors:** Xiao Xiao Tang, Hao Chen, Sidney Yu, Li Zhang, Michael J. Caplan, Hsiao Chang Chan

**Affiliations:** 1 Epithelial Cell Biology Research Center, School of Biomedial Sciences, Faculty of Medicine, the Chinese University of Hong Kong, Shatin, Hong Kong; 2 Department of Cellular and Molecular Physiology, Yale University, New Haven, Connecticut, United States of America; 3 Key Laboratory for Regenerative Medicine of the Ministry of Education of China, Guangzhou, China; Sun Yat-Sen University, China

## Abstract

The tight junctions (TJs), characteristically located at the apicolateral borders of adjacent epithelial cells, are required for the proper formation of epithelial cell polarity as well as for sustaining the mucosal barrier to the external environment. The observation that lymphocytes are recruited by epithelial cells to the sites of infection [1] suggests that they may play a role in the modulation of epithelial barrier function and thus contribute to host defense. To test the ability of lymphocytes to modulate tight junction assembly in epithelial cells, we set up a lymphocyte-epithelial cell co-culture system, in which Madin-Darby canine kidney (MDCK) cells, a well-established model cell line for studying epithelial TJ assembly [2], were co-cultured with mouse lymphocytes to mimic an infection state. In a typical calcium switch experiment, the TJ assembly in co-culture was found to be accelerated compared to that in MDCK cells alone. This accelaration was found to be mediated by AMP-activated protein kinase (AMPK). AMPK activation was independent of changes in cellular ATP levels but it was found to be activated by the pro-inflammatory cytokine TNF-α. Forced suppression of AMPK, either with a chemical inhibitor or by knockdown, abrogated the accelerating effect of lymphocytes on TJ formation. Similar results were also observed in a co-culture with lymphocytes and Calu-3 human airway epithelial cells, suggesting that the activation of AMPK may be a general mechanism underlying lymphocyte-accelerated TJ assembly in different epithelia. These results suggest that signals from lymphocytes, such as cytokines, facilitate TJ assembly in epithelial cells via the activation of AMPK.

## Introduction

Host defense against invading microbial pathogens at vairous epithelial surfaces relies both on the immune system and on an intact and protective epithelial cell layer. The surface epithelium of the mucosa forms a continuous barrier to a wide array of potentially harmful substances and microbial pathogens present in the lumen. Accordingly, maintaining barrier integrity represents a key issue in the defense capacity of the epithelium [3]. The tight junctions (TJs), characteristically located at the apicolateral borders of adjacent epithelial cells, are required for the proper formation of epithelial cell polarity as well as for the maintanence of the mucosal barrier. Furthermore, TJs function as the major barrier preventing the passage of ions and molecules through the paracellular pathway [4]. Thus, understanding the assembly of TJs and the mechanisms that regulate this process during infections are of great physiological importance.

It has been observed that during an infection lymphocytes are recruited by epithelial cells to the sites of infection [1], and they may play a role in host defense by modulating epithelial barrier function [5]. Some proinflammatory cytokines, such as TNF-á and IFN-γ, as well as certain virulence gene products from bacterial and viral pathogens, such as *Clostridium difficile* toxin A and rotavirus VP8 proteins, may induce epithelial TJ disassembly and disruption [6], [7], [8]. While many factors influence TJ formation in epithelial cells, the mechanism through which lymphocytes affect this process has not been studied.

Recently, it has been reported that AMP-activated protein kinase (AMPK) regulates TJ formation [9], [10]. AMPK was first discovered as a sensor of cellular energy status in all eukaryotic cells. It is activated in response to metabolic stresses such as muscle contraction or hypoxia, and modulated by hormones and cytokines that affect whole-body energy balance, such as leptin, adiponectin, resistin and ghrelin [11]. Once activated, it switches on catabolic pathways that generate adenosine triphosphate (ATP), while switching off ATP-consuming anabolic processes. AMPK exists as heterotrimeric complexes comprising a catalytic alpha-subunit and regulatory beta- and gamma-subunits. The binding of AMP to the gamma-subunit causes activation of the kinase by promoting phosphorylation at a threonine residue (Thr-172) on the alpha-subunit by the upstream kinase LKB1. High ATP content, a reflection of high cellular energy status, will antagonize the binding of AMP to the gamma-subunit, and this allows the system to act as a sensor of cellular energy status [12].

The present study investigates the effect of lymphocytes on epithelial TJ assembly in an epithelium-lymphocyte co-culture system, which mimics the infection state. Here we demonstrate that lymphocytes can accelerate/accentuate the assembly of TJs in epithelial cells and that AMPK is required during this process in an ATP-independent manner.

## Results and Discussion

### Lymphocytes facilitate co-cultured MDCK tight junction assembly

To establish an in vitro system that mimics the infection state in an epithelium, we co-culture lymphocytes with a widely used epithelial cell line, Madin-Darby canine kidney (MDCK) cells. We investigated whether the presence of lymphocytes affected the assembly of TJ by MDCK cells. TJ assembly can be manipulated by changing the extracellular concentration of calcium. Extracellular calcium is essential for the assembly of cell junctions [13], [14]. Depletion of calcium from the medium causes the TJ protein zonula occludens-1 (ZO-1) to translocate from the cell periphery to the cytoplasm, and readdition of calcium to the medium (a maneuver referred to as a “calcium switch”) triggers junction assembly and cell polarization [2], [15].

**Figure 1:**
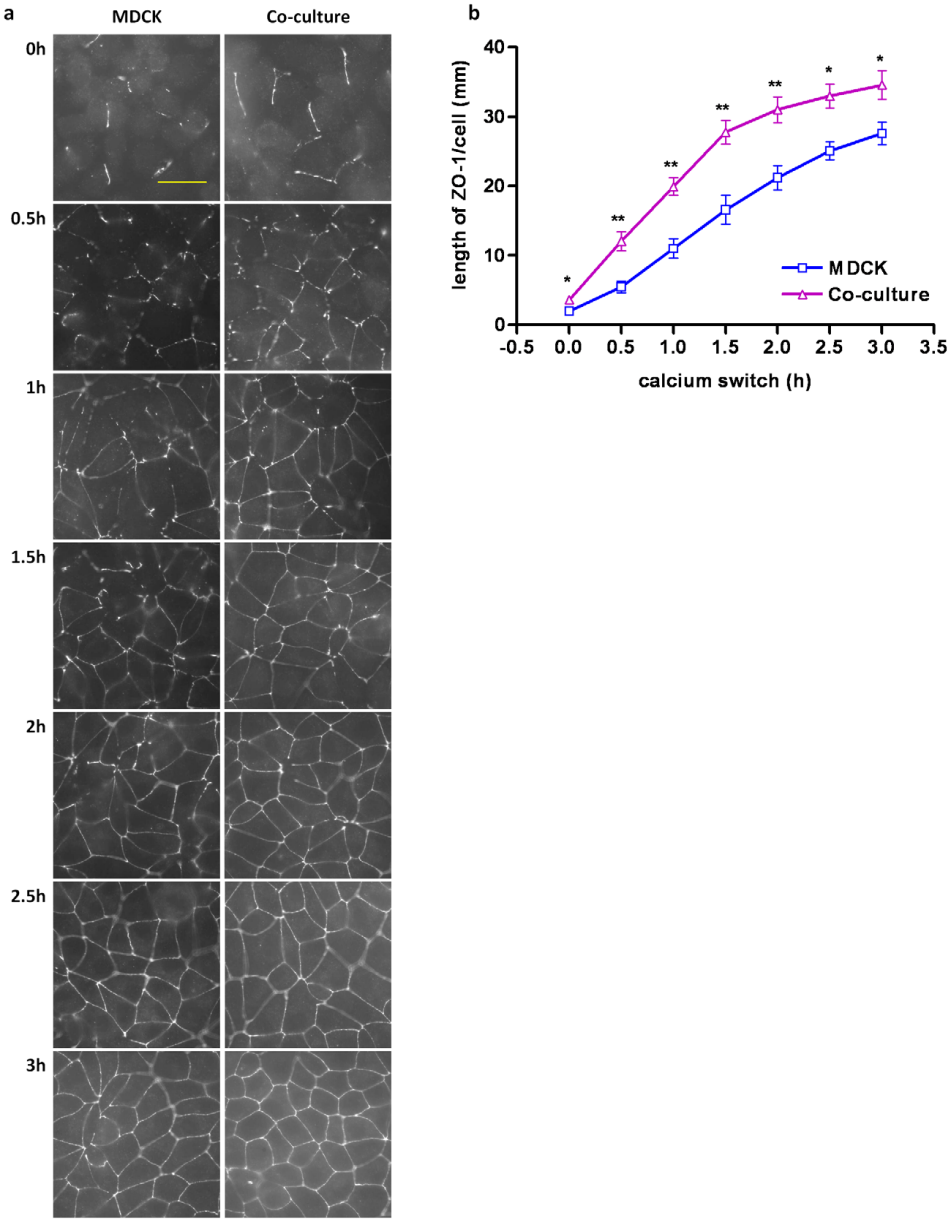
Lymphocyte co-culture facilitates MDCK tight junction assembly. (a) Representative pictures of ZO-1 relocation to cell–cell junctions in MDCK cells cultured alone and co-cultured with lymphocytes at various time points (0h, 0.5h, 1h, 1.5h, 2h, 2.5h, 3h) after calcium switch. (Scale bar: 30 µm). (b) Quantification of ZO-1 relocation to cell–cell junctions (length of ZO-1/cell (µm)) in MDCK cells cultured alone and co-cultured with lymphocytes at various time points (0h, 0.5h, 1h, 1.5h, 2h, 2.5h, 3h) after calcium switch. The asterisks denote significant differences detected in the presence vs. the absence of lymphocytes by Student's *t* test (*, p<0.05; **, p<0.01).

The relocation of ZO-1 to cell–cell junctions represents an important step in the initiation of TJ assembly [9]. To evaluate the rate of calcium-induced TJ assembly, therefore, we monitored the time course of the ZO-1 relocation to the cell–cell junctions after the readdition of calcium in the MDCK cells with or without lymphocytes. At various time points measured, we found that ZO-1 relocation is accelerated significantly in the MDCK cells co-cultured with lymphocytes during calcium switch (**Fig. 1a, b**). Significant increase in the length of linear membrane-associated patches of ZO-1 per cell was observed in lymphocyte co-cultured MDCK cells compared to that observed in MDCK cells alone (**Fig. 1a, b**). This result indicates that lymphocytes can influence aspects of the TJ assembly in MDCK cells.

### AMPK is activated in the epithelial-lymphocyte co-culture

**Figure 2:**
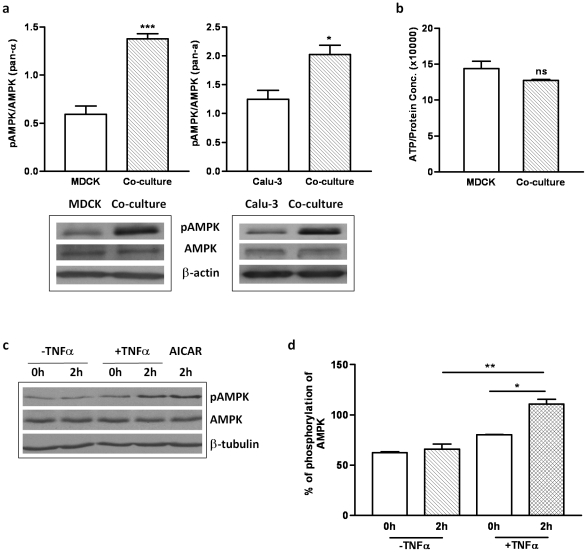
AMPK is activated in the epithelium-lymphocyte co-culture and by TNF-α. (a) Western blot results showing increased level of AMPK phosphorylation in MDCK-lymphocyte co-culture and Calu-3-lymphocyte co-culture as compared to MDCK and Calu-3 cultured alone, respectively. Total AMPK remained unchanged, indicating that AMPK is activated in these co-cultures, with beta-actin used as loading control. The corresponding statistical analysis is on the right panel (*, p<0.05; ***, p<0.001). (b) ATP assay showing no significant changes of cellular ATP levels in MDCK-lymphocyte co-culture vs. MDCK cells alone (‘ns’ stands for no significance). Cell lysates from MDCK cells with and without lymphocytes were subjected to an ATP assay. The ATP contents were normalized to the total protein amount. Data were from three experiments. (c) Lysates prepared from MDCK cells maintained in α-MEM in the absence or presence of TNF-α were blotted with the indicated antibodies. Treatment with AICAR served as the positive control for AMPK activation. (d) The activated AMPK was represented as a relative ratio to total AMPK. The phosphorylation of AMPK is significantly increased by exposure for 2 hours to TNF-α treated, as assessed by *Student's test* (**, p<0.01).

AMPK was reported to be involved in TJ assembly [9], [10] and it has also been implicated in inflammatory responses [16]. We decided, therefore, to examine whether AMPK is involved in the accelerating effect of lymphocytes on TJ assembly. As a first step, we investigated whether AMPK is activated in MDCK cells by Western blot. As shown in **Fig. 2a**, phosphorylation of AMPK at the Thr172 residue on the alpha-subunit was drastically increased in co-culture compared with MDCK alone whereas total AMPK remained unchanged as shown in a blot probed with AMPK (pan-α) antibody that recognizes both phosphorylated and unphosphorylated AMPK. This result indicates that lymphocyte co-culture creates the potential for involvement of AMPK in TJ assembly of MDCK cells. To determine whether AMPK activation is a general phenomenon that occurs when epithelial cells are co-cultured with lymphocytes, we also examined the level of AMPK activation in another epithelial cell line of different origin, the human airway epithelial cell line, Calu-3. Similar to what has been observed in MDCK cells,, the level of AMPK Thr-172 phosphorylation increased in Calu-3-lymphocyte co-culture, as shown in **Fig. 2a**, suggesting that AMPK activation may be a general mechanism underlying lymphocyte-accelerated TJ assembly in different epithelia.

AMPK is a critical sensor of ATP levels in cells [11]. Thus, the activation of AMPK might be attributed to changes in cellular energy status in the co-culture that can possibly be induced by the lymphocytes, since the calcium switch does not itself produce substantial changes in cellular ATP levels [9]. To assess this possibility, we examined the cellular ATP levels in MDCK cells in the presence or absence of lymphocytes. No significant changes were observed in cellular ATP levels in MDCK cells cultured with or without lymphocytes (**Fig. 2b**), indicating that AMPK activation in the co-culture was not due to ATP deficiency.

The most likely candidates for the lymphocyte factors that trigger AMPK activation are cytokines, which are known to be involved in epithelium-lymphocyte crosstalk. There is growing evidence demonstrating AMPK activation in response to cytokines [17], [18], [19]. Indeed, we found that incubating MDCK cells with TNF-α in the absence of lymphocytes could also result in activation of AMPK as revealed by a two-fold increase of Thr-172 phosphorylation (Figure 2c and 2d). This result suggests that cytokines such as TNF-α, which can be released upon epithelium-lymphocyte intereaction [20], may activate AMPK in epithelial cells to accelerate TJ assembly. Interestingly, in our previously study [5], when lymphocytes and epithelial cells were co-cultured in separate compartments the barrier-enhancing effect was not observed, indicating that cell-cell interaction is required. However, that does not necessarily exclude the possibility that cytokines alone can enhance the TJ formation. In fact, we have observed increases in cytokines release in a coculture of endometrial epithelial cells and lymphocytes and upregulation of ZO-1 expression in epithelial cell culture alone by TNF-α or IL-β [20]. Taken together, it suggests that the release of cytokines will require cell-cell contact between lymphocytes and epithelial cells and that the released cytokines such as TNF-α may be responsible for mediating the barrier-enhancing effect or acceleration of TJ formation.

### Lymphocyte-accelerated tight junction assembly is mediated by AMPK

**Figure 3:**
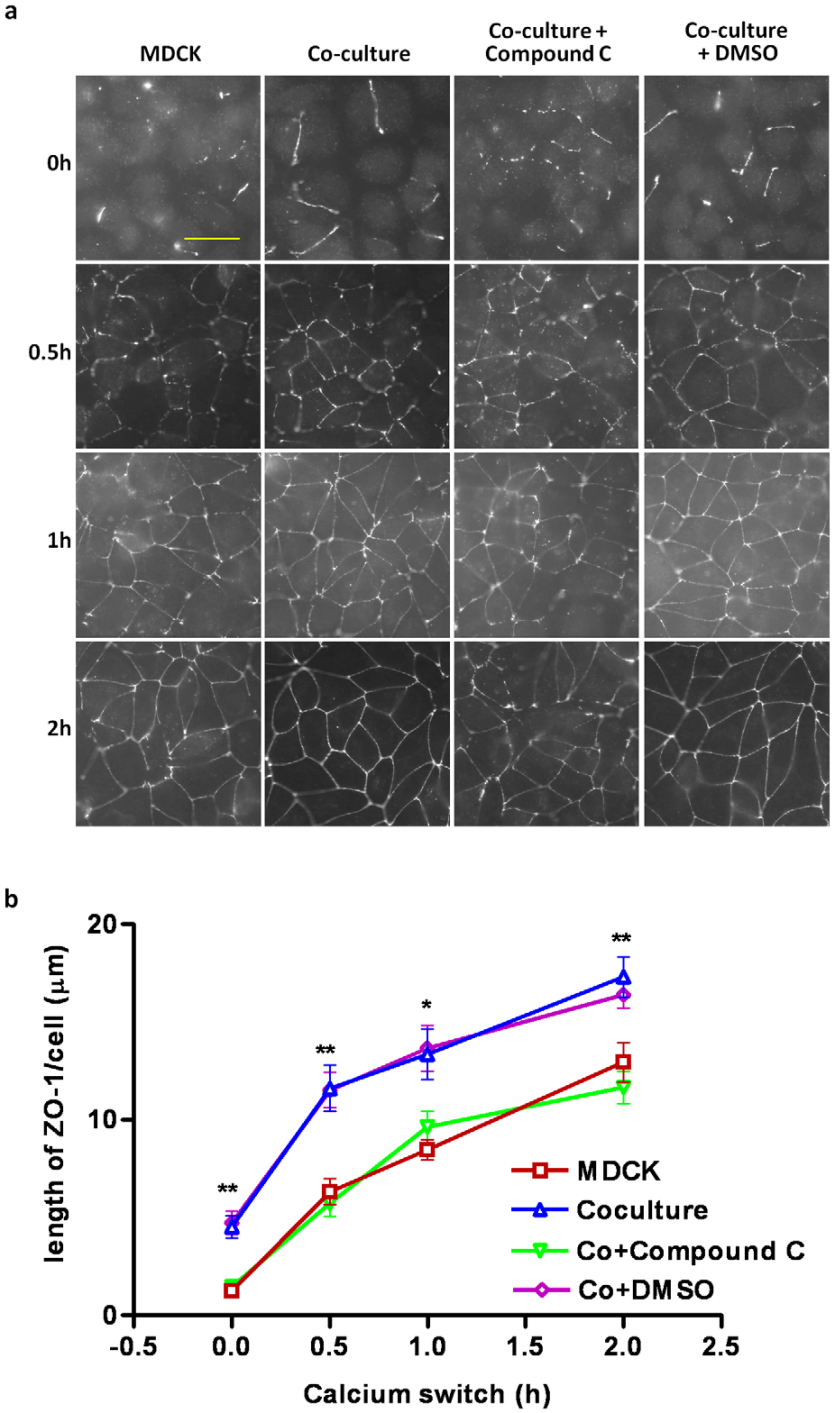
AMPK inhibitor, Compound C, abolishes the lymphocyte-facilitation of tight junction assembly. (a) Representative pictures of ZO-1 relocation to cell–cell junctions in MDCK cells cultured alone and co-cultured with lymphocytes, with or without the AMPK inhibitor, Compound C, (A. MDCK; B. Co-culture; C. Co-culture+Compound C; D. Co-culture+DMSO) at various time points (0h, 0.5h, 1h, 2h) after calcium switch. (Scale bar: 30 µm). (b) Quantification of ZO-1 relocation to cell–cell junctions (length of ZO-1/cell (µm)) in MDCK cells cultured alone and co-cultured with lymphocytes, with or without AMPK inhibitor, Compound C, at various time points (0h, 0.5h, 1h, 2h) after calcium switch. The asterisks denote significant differences detected in (C) Co-culture+Compound C vs. (D) Co-culture+DMSO by Student's *t* test (*, p<0.05; **, p<0.01).

**Figure 4:**
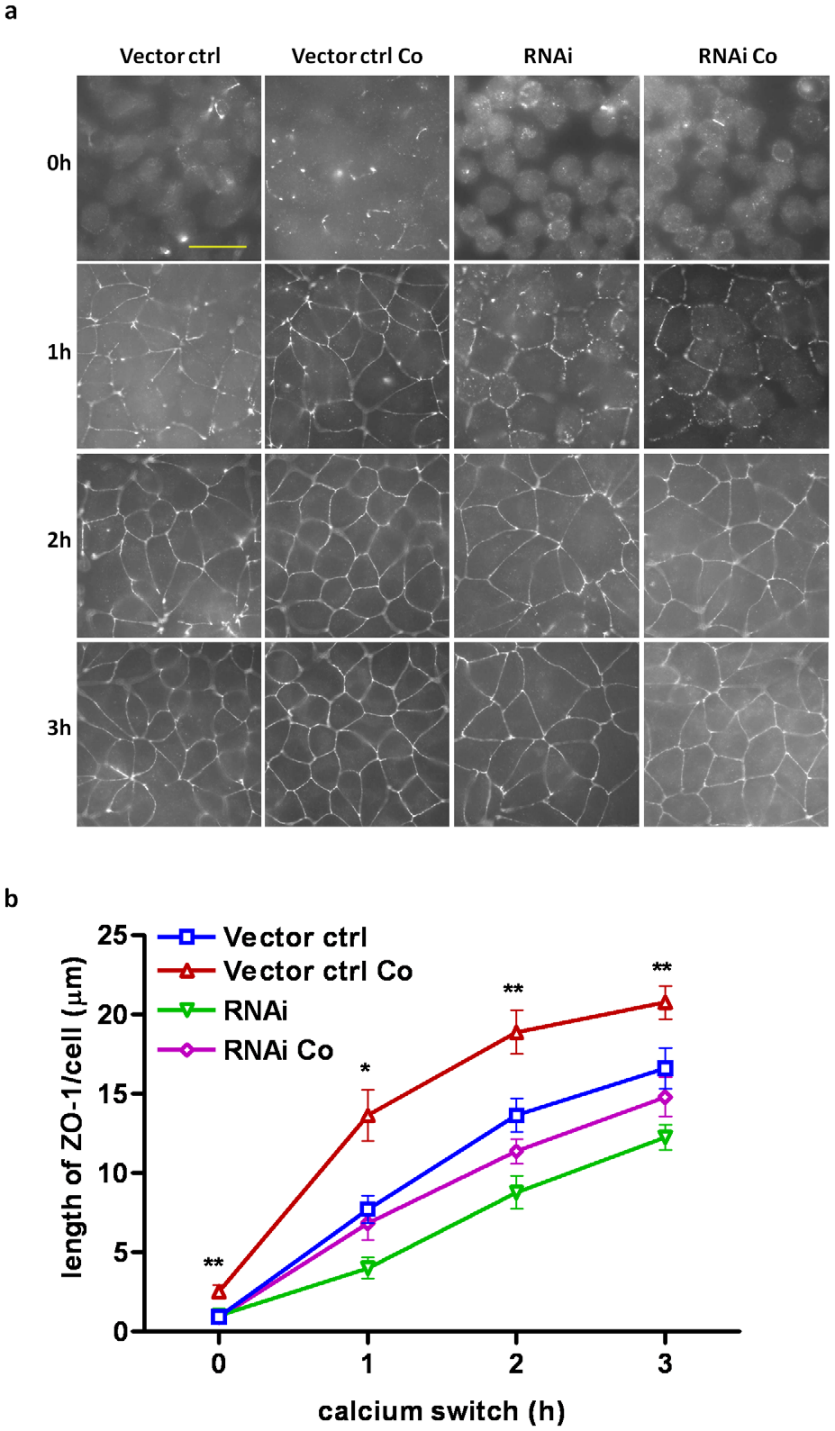
shRNA-mediated AMPK knockdown inhibits the lymphocyte-facilitation of tight junction assembly. (a) Representative pictures of ZO-1 relocation to cell–cell junctions in (A) Vector control cells; (B) Co-cultured vector control cells; (C) shRNA AMPK cells and (D) Co-cultured shRNA AMPK cells at various time points (0h, 1h, 2h, 3h) after calcium switch. (Scale bar: 30 µm). (b) Quantification of ZO-1 relocation to cell–cell junctions (length of ZO-1/cell (µm)) in culture conditions as indicated in (a) at various time points (0h, 1h, 2h, 3h) after calcium switch. The asterisks denote significant differences detected in (B) co-cultured vector control cells vs. (D) co-cultured shRNA AMPK cells by Student's *t* test (*, p<0.05; **, p<0.01).

To further investigate whether the lymphocyte-accelerated TJ assembly was due to the activation of AMPK observed in the co-culture, we used the AMPK inhibitor, Compound C, as well as MDCK cells subjected to shRNA-mediated AMPK knockdown in the calcium switch model. Compound C abolished the lymphocyte-accelerated TJ assembly in the co-culture as measured by the extent of ZO-1 localization to the TJ after calcium switch (**Fig. 3a, b**), indicating that AMPK activation is critical to the lymphocyte-acceleration of TJ assembly. To further confirm the important role of AMPK in this process, MDCK cells expressing shRNA directed against the AMPK α1 subunit protein and a vector control cell line were tested for their rate of TJ assembly. The α1 isoform is the predominant AMPK α isoform expressed in MDCK cells [9]. As illustrated in **Fig. 4a, b**, the TJ assembly rate in the co-cultured vector control cells was significantly higher than that in the vector control cells alone, suggesting that the transfected control vector does not impair the lymphocyte-accelerated TJ assembly process in the MDCK cells. However, the TJ assembly rates in AMPK shRNA cells failed to elicit a lymphocyte-accelerated TJ assembly in the co-cultured condition, confirming the requirement for AMPK in this process. In short, forced suppression of AMPK activity, either through a chemical inhibitor or through shRNA-mediated expression knockdown, inhibited the lymphocyte-accelerated TJ formation.

Taken together, these data demonstrate a novel and interesting physiological process through which lymphocytes accelerate epithelial TJ assembly. In addition, this process is mediated by the activation of AMPK in the target epithelial cells. This process may contribute to the host defense against invading microbials by helping to maintain the protective barrier function of TJs during infections. The involvement of AMPK in TJ assembly is intriguing because AMPK sits at a unique position as an energy sensor that interfaces with diverse signaling molecules [21]. However, the present results indicate that the activation of AMPK required for lymphocyte-accelerated TJ assembly is not dependent on cellular ATP levels but may be mediated by cytokines. Interestly, a number of the pathways that activate AMPK, including osmotic stress and changes in cytosolic calcium, also do so through energy-metabolism independent pathways [22], supporting an important role of the non-ATP-dependent activation of AMPK in various cellular responses. In the future it will be import to determine the exact mechanism through which lymphocytes activate AMPK to facilitate epithelial TJ assembly upon infection.

## Materials and Methods

### Animals

Adult male C57BL/6J mice from Charles River Laboratories were used for peripheral blood collection.

### Cells

Type II Madin-Darby canine kidney (MDCK) cells [9], [10] were used in the experiments. AMPK α1 subunit knockdown MDCK was also generated. Sequences targeting the canine AMPK α1 subunit was subcloned into pLH1, a derivative of the pSUPER plasmid. Plasmids were transfected with pVSVG into HEK-G2 cells for lentivirus packaging. Subconfluent MDCK cells were then infected with the resultant lentivirus. Selection and maintenance of stable MDCK cell clones were performed in α-MEM media containing 4 µg/mL puromycin (Sigma). Clones were screened for reduced expression levels of AMPK α1 subunit by Western blot analysis. An empty pLH1 plasmid was also packaged into lentivirus and a pooled control MDCK cell line was established by infection with this lentivirus. The sequence chosen for targeting AMPK α1 subunit was 5′-GCAGAAGTTTGTAGGGCAATT-3′.

Peripheral blood lymphocytes (PBL) were isolated from whole blood using gradient centrifugation. Fresh peripheral blood was collected from anesthetized adult mice with heart puncture technique using a 25×g 5/8″gauge needle and placed in anti-coagulating EDTA KE tubes to avoid clotting. The blood was diluted 1∶1 with balanced salt solution. The diluted blood was carefully layered on 3ml Ficoll in a centrifuge tube and centrifuged without brake at 400 g for 30–40 minutes at 18–20°C. (The diluted blood sample should not be mixed with the Ficoll-Paque PLUS when layering the sample.). The upper layer was drawn off using a clean Pasteur pipette, leaving the lymphocyte layer undisturbed at the interface. The lymphocyte layer was carefully collected and transferred to a clean centrifuge tube using a clean Pasteur pipette. The pelleted red blood cells (RBCs) and granulocytes were discarded. PBL were washed in an equal volume of 1× PBS and pelleted by centrifuging at 400 g for 5 minutes. The remaining traces of RBCs were further removed by adding 2 ml of sterile distilled water for 30 seconds to lyse the RBCs. An equal volume of 2× PBS was quickly added to equilibrate osmotic pressure. PBL were pelleted at 400 g for 5 minutes.

### Calcium Switch

MDCK cells were grown in MEM Alpha until confluency. Cells were then rinsed with Ca^2+^-free S-MEM and incubated in S-MEM supplemented with 5% dialyzed FBS for 16 h before being switched back to MEM Alpha for the indicated times [9], [10].

### MDCK-lymphocyte co-culture

MDCK cells were grown in MEM Alpha supplemented with 10% fetal bovine serum and 1% penicillin-streptomycin. The cells were incubated in a humidified atmosphere containing 5% CO_2_ at 37°C. MDCK cells were co-cultured with PBL according to the methods previously described [23].

### Immunofluorescence and Quantification of ZO-1 Staining

Cells on coverslips were fixed in cold methanol and then permeabilized in goat serum dilution buffer (GSDB) (16% goat serum/20 mM Na_3_PO_4_, pH 7.4/450 mM NaCl/0.3% Triton X-100). Cells were blocked with GSDB for 30 min and incubated for 1 h with anti-ZO-1 (Zymed), followed by incubation with Alexa Fluor 488-conjugated anti-rabbit IgG (Molecular Probes). Cells were visualized on an Axiophot microscope (Carl Zeiss). Contrast and brightness settings were chosen so that all pixels were in the linear range.

To quantify the average ZO-1 length per cell, four fields were randomly selected from each coverslip, and the total length of ZO-1 at cell junctions in each field was outlined manually, followed by measurement using Image J software (National Institutes of Health). Cell numbers were counted for each field by using propidium iodide (Molecular Probes) staining to reveal nuclei, and the mean ZO-1 length per cell was calculated.

### ATP Assay

ATP concentrations were determined by using the FL-AA Bioluminescent assay kit (Sigma). Briefly, cells were lysed with 0.5 ml water and sonicated for 30 seconds. The cells were scraped off the plate and transferred quantitatively into microfuge tubes. This was followed by boiling in a water bath for 3 minutes to inactivate any ATP hydrolytic activity present in the cell lysates. All samples were stored frozen at −80°C and aliquots were removed for analysis of ATP as described below.

Frozen samples were thawed and centrifuged at 15,000 rpm for 10 minutes in a Beckman (Beckman Coulter, Fullerton, CA, USA) microfuge at 4°C. Supernatant (25 µl) was used for ATP determination following the procedure provided in the Sigma FL-AA Bioluminescent assay kit. Essentially, the assay mixture contained 25 µl of cell extract and 100 µl of ATP assay mix (FL-AAM), which had been diluted 250-fold in adenosine 5′triphosphate (ATP) assay mix dilution buffer (FL-AAB) solution according to the instructions from the suppliers. Measurements of the chemiluminescence of the luciferin-luciferase reaction were made in a Beckman luminitor. This is a reliable and accurate assay with the added advantage of permitting the measurment of multiple samples with ease.

### Western Blot Analysis

Cells were lysed on ice in lysis buffer (100mM NaCl, 1mM EDTA (pH 8), 50mM Tris (pH 7.5), 1% Triton X-100, 1 tablet protease inhibitors per 50ml) and cell lysates were obtained by centrifugation at 14000 rpm for 20 min at 4°C. After the protein concentration was determined, the cell lysates containing equal amounts of protein were loaded onto SDS-PAGE. Blots were incubated with pAMPK (T172) (Cell signaling, MA, USA), AMPK (pan-α) (Upstate Biotechnology, Lake Placid, NY, USA) and beta-actin (Abcam, Cambridge, MA, USA) antibodies. Enhanced chemiluminescence (ECL; Amersham Biosciences, UK) was visualized by film development.

Evaluation of the level of phosphorylated AMPK that was induced in response to TNF-α was examined in MDCK cells, which were seeded in 6-well plate at 5×10^5^ per well and incubated for overnight at 37°C and 5% CO_2_. The cells were then treated with 10ng/ml TNF-α or 2mM AICAR (as the positive control) for 2 hours. Cells were lysed with kinase lysis buffer as previous described [9] and western blotting was performed to evaluate the phosphorylation of AMPK.

### Ethics

The experimental protocols used in the study were approved by the Animal Ethics Committee of the Chinese University of Hong Kong (06/048/MIS).
